# Ancient mitogenomes reveal a high maternal genetic diversity of Pleistocene woolly rhinoceros in Northern China

**DOI:** 10.1186/s12862-023-02168-0

**Published:** 2023-09-26

**Authors:** Junxia Yuan, Guojiang Sun, Bo Xiao, Jiaming Hu, Linying Wang, Taogetongqimuge, Lei Bao, Yamei Hou, Shiwen Song, Shan Jiang, Yong Wu, Dong Pan, Yang Liu, Michael V. Westbury, Xulong Lai, Guilian Sheng

**Affiliations:** 1 State Key Laboratory of Biogeology and Environmental Geology, China University of Geosciences, Wuhan, 430078 China; 2 Bioarchaeology Laboratory, Jilin University, Changchun, 130012 China; 3 Faculty of Materials Science and Chemistry, China University of Geosciences, Wuhan, 430078 China; 4 School of Earth Science, China University of Geosciences, Wuhan, 430074 China; 5 Wushen Banner Museum, Ordos, 017399 China; 6 Ordos Institute of Cultural Relics and Archaeology, Ordos, 017010 China; 7 Key Laboratory of Vertebrate Evolution and Human Origins, Institute of Vertebrate Paleontology and Paleoanthropology, Chinese Academy of Sciences, Beijing, 100044 China; 8 School of Environmental Studies, China University of Geosciences, Wuhan, 430078 China; 9 The Third Geological and Mineral Exploration Institute of Gansu Bureau of Geology and Mineral Resources, Lanzhou, 730050 China; 10 Palaeontological Fossil Conservation Center, Qinggang County, Suihua, 151600 China; 11 School of Sociology & Anthropology, Sun Yat-sen University, Guangzhou, 510275 China; 12 Globe Institute, University of Copenhagen, Copenhagen, 1353 Denmark; 13 Present Address: College of Earth and Environmental Science, Lanzhou University, Lanzhou, 730099 China

**Keywords:** Woolly rhinoceros, Maternal diversity, Northern China, Dispersal, Pleistocene

## Abstract

**Background:**

Woolly rhinoceros (*Coelodonta antiquitatis*) is a typical indicator of cold-stage climate that was widely distributed in Northern Hemisphere during the Middle-Late Pleistocene. Although a plethora of fossils have been excavated from Northern China, their phylogenetic status, intraspecific diversity and phylogeographical structure are still vague.

**Results:**

In the present study, we generated four mitogenomes from Late Pleistocene woolly rhinoceros in Northern China and compared them with published data. Bayesian and network analyses indicate that the analyzed individuals contain at least four maternal haplogroups, and Chinese samples fall in three of them. One of our samples belongs to a previously unidentified early diverging clade (haplogroup D), which separated from other woolly rhinoceros around 0.57 Ma (95% CI: 0.76–0.41 Ma). The timing of this clade’s origin coincides with the first occurrence of woolly rhinoceros, which are thought to have evolved in Europe. Our other three samples cluster in haplogroup C, previously only identified from one specimen from Wrangel Island (ND030) and initially considered to be an isolated clade. Herein, our findings suggest that ND030 is likely descended from a northward dispersal of the individuals carrying haplogroup C from Northern China. Additionally, Chinese woolly rhinoceros specimens exhibit higher nucleotide diversity than those from Siberia.

**Conclusion:**

Our findings highlight Northern China as a possible refugium and a key evolution center of the Pleistocene woolly rhinoceros.

**Supplementary Information:**

The online version contains supplementary material available at 10.1186/s12862-023-02168-0.

## Introduction

Woolly rhinoceros (*Coelodonta antiquitatis*), an extinct rhino of the genus *Coelodonta*, was particularly well adapted to cold climates and widely distributed in Northern Hemisphere during the Middle to Late Pleistocene (about 0.46 − 0.012 million years ago, Ma), ranging from Western Europe to Northeastern Siberia [1–3]. As early as tens of thousands of years ago, woolly rhinoceros was closely related with human and many images have been found in some caves [4, 5]. As one of the iconic elements of “*Mammuthus*-*Coelodonta* faunas”, woolly rhinoceros has been investigated morphologically in numerous previous studies [6–9]. It is generally accepted that the morphometric parameters of Late Pleistocene Eurasian woolly rhinoceros all fall within the range of intraspecific variation [7, 8]. By analyzing morphological characteristics of the Late Pleistocene woolly rhinoceros fossils from Northern China and Siberia, Zhou [6] suggested that Chinese individuals are the descendants of Siberian relatives.

Ancient DNA has greatly improved our insights into the phylogenetic position of woolly rhinoceros in the family Rhinocerotidae [10–16]. Genetic data supports that the extinct woolly rhinoceros is more closely related to extant Sumatran rhinoceros (*Dicerorhinus sumatrensis*) than to other four living rhinos, that is black rhinoceros (*Diceros bicornis*), white rhinoceros (*Ceratotherium simum*), Indian rhinoceros (*Rhinoceros unicornis*), and Javan rhinoceros (*Rhinoceros sondaicus*), respectively [10–12]. Among the extinct rhinos, woolly rhinoceros exhibits a closer relationship to Merck’s rhinoceros (*Stephanorhinus kirchbergensis*) than to Siberian unicorn (*Elasmotherium sibiricum*) [15]. With regard to intraspecific genetic diversity of woolly rhinoceros, a recent mitochondrial study detected three maternal lineages from Siberian specimens [14], in which one sample ND030 (39,652 ± 952 cal yBP) from Wrangel Island forms a genetically distinct clade that separates from other individuals excavated from the same or adjacent region. So far, little is known about its geographic origin and other past distribution of this enigmatic woolly rhinoceros clade.

**Fig. 1:**
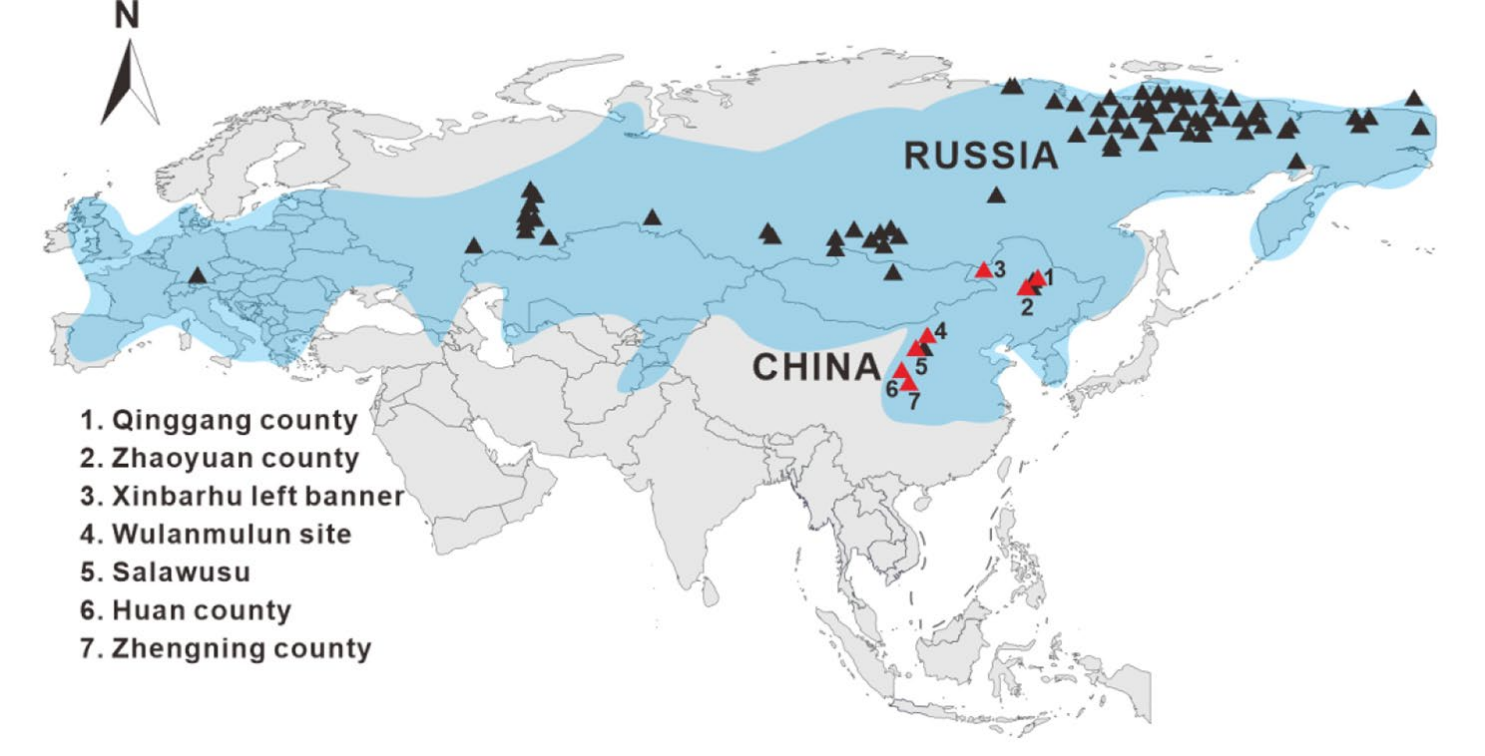
Sampling sites of woolly rhinoceros. Black triangles indicate the locations of woolly rhinoceros samples used for molecular analyses in previous studies [12, 14, 20–22]. Red triangles indicate the locations of our samples in the present study. Blue shadow area stands for the past distribution range of woolly rhinoceros in Northern Eurasia [15]

Members of the genus *Coelodonta* experienced a long-term history in China [2, 6, 17]. The earliest known representative of this genus, *Coelodonta thibetana* (~ 3.7 Ma), inhabited Zanda County, Tibet Autonomous Region, China [2]. During the Late Pleistocene, woolly rhinoceros was widely distributed in Northern China [6, 18, 19]. Unfortunately, so far, only several short mitochondrial DNA fragments have been retrieved from Chinese specimens, preliminary assessments indicate that Chinese individuals show a close relationship to their counterparts from Siberia [12]. In this study, we generated four mitochondrial genomes from 16 Late Pleistocene woolly rhinoceros individuals collected from Northern China (Fig. 1). Combining these sequences with previous published data to explore the maternal phylogenetic status of available Chinese woolly rhinoceros, we refined the intraspecies divergence of this species and estimated the divergence times of different haplogroups. We also calculated nucleotide diversity of Late Pleistocene woolly rhinoceros specimens from different regions in Asia. In combination, our findings provide new sights into the molecular evolution of Chinese woolly rhinoceros and contribute to a better understanding of past dispersal events of this extinct rhino.

## Results

### Mitochondrial genomes

Four mitochondrial genomes were generated from Late Pleistocene woolly rhinoceros samples from Northern China, with callable bases between 12,660 and 16,233 base pairs (bp) and mean depths between 3.31 and 20.60 folds (Table S1). The DNA fragments exhibit increased C→T deamination rates at the 5’ ends (Figs. S1 and S2) and short average lengths (56–74 bp) (Table S1), which are consistent with the characteristics of ancient DNA. The age of CADG739 was dated at 44,535–43,349 cal yBP (the median calibrated age: 43,942 years ago), and the ages of the other three samples (CADG744, CADG900 and CADG912) are beyond the calibration range (48,000 cal yBP) (Table S1). We estimated these three samples at the median ages 62,453, 51,721, and 59,810 years ago, respectively, using molecular clock analysis in BEAST (Table S2).

### Bayesian tree

**Fig. 2:**
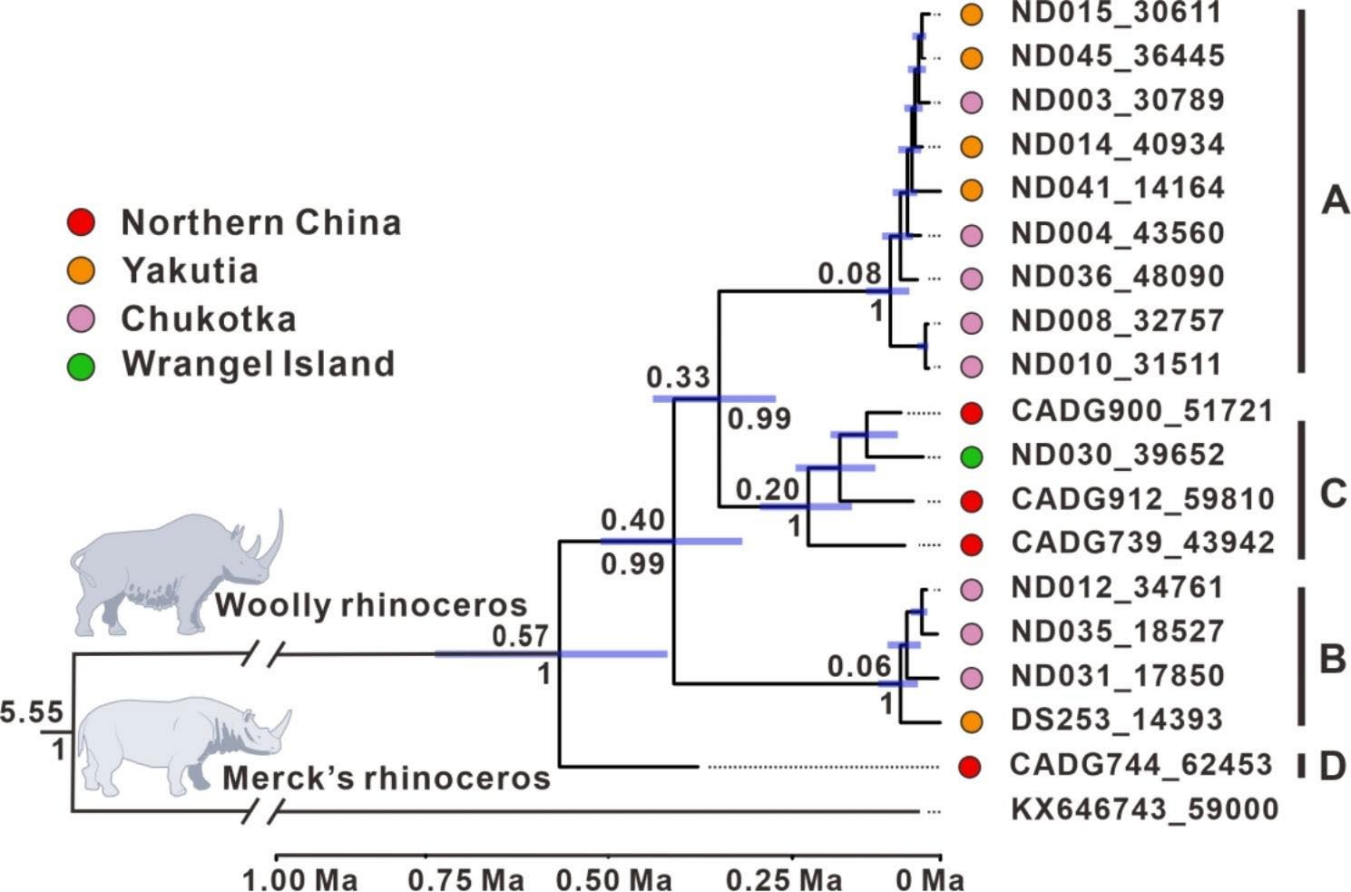
Bayesian phylogeny of woolly rhinoceros using 11,704 bp homologous mitogenomic sequences. A timescale is placed at the bottom of the tree. Labels on tips of branches correspond to the geographical regions, sample codes and ages (median calibrated radiocarbon or molecularly estimated ages). Labels above nodes indicate the divergence times with blue bars showing the 95% credibility intervals (CI). Numbers below the nodes represent the posterior values

A Bayesian tree based on 11,704 bp of the mitogenomes suggests that the analyzed woolly rhinoceros individuals are divided into four well-supported haplogroups (Fig. 2). Haplogroups A and B are represented by samples from Yakutia and Chukotka in Siberia, which are consistent with a previous study [14]. Three out of four Northern Chinese samples, i.e., CADG739, CADG900, and CADG912, cluster in haplogroup C, previously represented by only one individual (ND030) from Wrangel Island [14]. Our other sample (CADG744) stands out as a newly identified mitochondrial haplogroup (D) basal to all other woolly rhinoceros with strong support (100% posterior probability).

To estimate the divergence times of the identified woolly rhinoceros haplogroups, we used root and tip-dating methods to calibrate the Bayesian tree (Fig. 2), obtaining a mean mutation rate of the mitogenomes of 6.90 × 10^− 9^ substitutions/site/year (95% CI: 5.56 × 10^− 9^–8.47 × 10^− 9^ substitutions/site/year), less than that of previous estimation [14]. The divergence time between Merck’s rhinoceros and woolly rhinoceros was estimated to 5.55 Ma (95% CI: 4.64–6.56 Ma), which is close to the previous estimations conducted by Margaryan et al. [22] using mitogenomes. The time of the most recent common ancestor (tMRCA) of the analyzed woolly rhinoceros samples is estimated to 0.57 Ma (95% CI: 0.76–0.41 Ma). The split between haplogroup B and haplogroups A/C occurred at 0.40 Ma (95% CI: 0.51–0.30 Ma), and the separation between haplogroups A and C is estimated to 0.33 Ma (95% CI: 0.43–0.25 Ma), which are older than those of estimations by Lord et al. [14]. Additionally, our estimate of the tMRCA of haplogroup C is 0.20 Ma (95% CI: 0.27–0.13 Ma).

### Median-joining network analysis

**Fig. 3:**
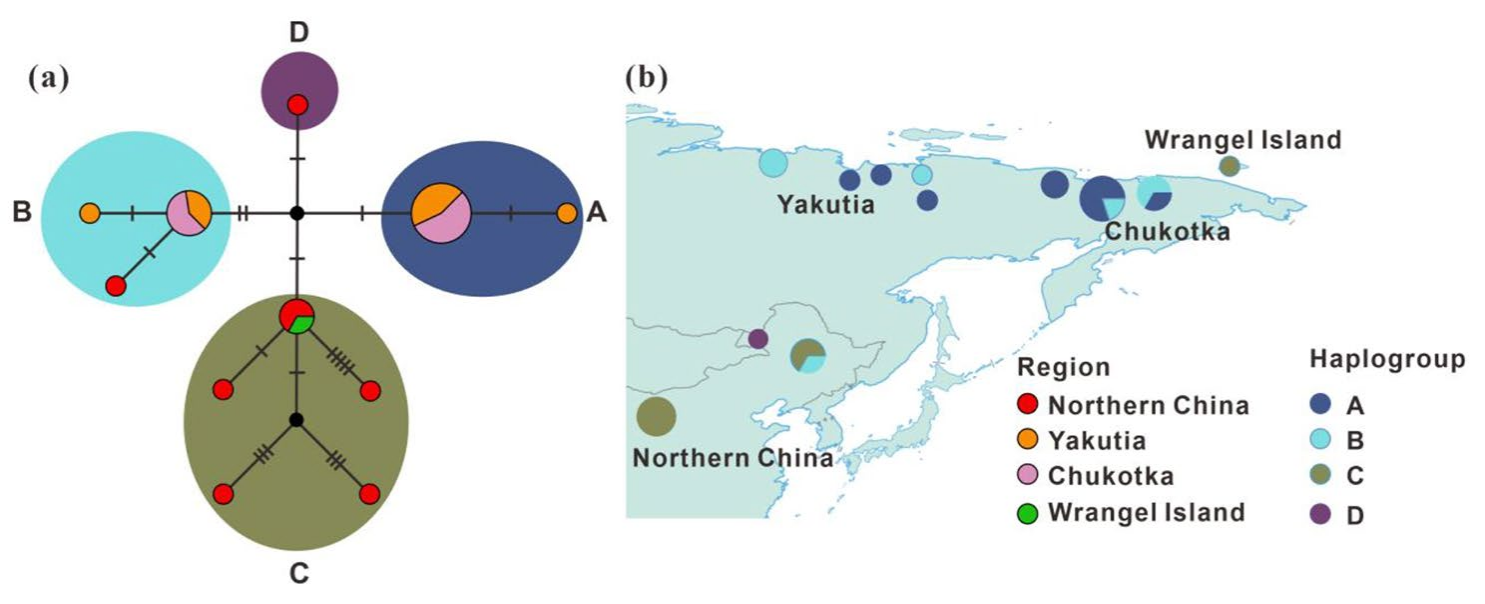
Median-joining network and phylogeographic distribution of woolly rhinoceros. **(a)** Median-joining network based on 563 bp *cyt b* sequences of woolly rhinoceros from Northern China, Yakutia, Chukotka and Wrangel Island. Each circle represents an independent haplotype and black nodes are missing haplotypes. Circle colors indicate the geographical regions. Hatch marks represent the number of mutational sites between two adjacent haplotypes. Detailed information of haplotype is listed in Table S2. **(b)** Geographical distribution of the maternal haplogroups revealed by the median-joining network analysis. Circle colors indicate the haplogroups. The sizes of the circles in both **(a)** and **(b)** are proportional to the number of the sample of given haplotype (**(a)**: Maximum = 10; **(b)**: Maximum = 5)

To fully investigate the genetic diversity of Chinese woolly rhinoceros, we redrew more sequences of woolly rhinoceros from Northern China and carried out a median-joining network analysis based on 563 bp partial *cyt b* gene. We generated an identical pattern to our BEAST analysis, with four maternal haplogroups identified (Fig. 3; Table S2). Including four Chinese samples from a previous study [12], a total of eight Chinese individuals form seven haplotypes, which fall into three haplogroups (B, C and D). One sample (HS12) from Yuan et al. [12] clusters with Siberian individuals in haplogroup B. Six out of eight Chinese samples group in haplogroup C. The sample CADG744 belongs to a distinct lineage, in agreement with Bayesian analysis based on 11,704 bp mitogenomic sequences.

### Nucleotide diversity

Nucleotide diversity estimation reveals that woolly rhinoceros samples from Northern China possess relatively higher nucleotide diversity than those from Yakutia and Chukotka (including the Wrangel Island individual ND030) (Table 1).

**Table 1:** Nucleotide diversity of woolly rhinoceros from Northern China, Yakutia and Chukotka based on 11,609 bp mitogenomes

Geographical region	Sample number (n)	Nucleotide diversity (π)
Northern China	4	0.003962
Yakutia, Russia	8	0.002310
Chukotka, Russia	9	0.002881

## Discussion

### Deeply divergent woolly rhinoceros clade from Northern China

In the current study, both Bayesian and network analyses indicate that there are four distinct maternal clades in the analyzed woolly rhinoceros. Notably, an early separating maternal clade (i.e., haplogroup D) is newly detected in our phylogenetic analyses (Figs. 2 and 3). Although this ancient woolly rhinoceros clade is solely represented by only one sample in this study, several deeply divergent mitochondrial or nuclear clades have been identified from other Late Pleistocene large herbivores and carnivores in Northern China, e.g., aurochs (*Bos primigenius*) [23], steppe bison (*Bison priscus*) [24], cave hyenas (*Crocuta crocuta ultima*) [25, 26], and tiger (*Panthera tigris*) [27]. These distinct ancient clades from different species experienced a long evolutionary history in the same area, which suggests that Northern China might have represented a refugium for some Quaternary mammals. Therefore, we suppose that this region will be one of the hot-spots in investigating long-term occupation, migration and evolution of mammals under pressure of Quaternary climate and environment changes.

Our finding raises another fascinating question regarding to the origin and dispersal of woolly rhinoceros haplogroup D. According to fossil remains, paleontologists have suggested that the earliest known representative of the genus *Coelodonta*, *Coelodonta thibetana* (~ 3.7 Ma), originated in Zanda Basin, Western China, and it evolved into *Coelodonta Nihowanensis* (~ 2.5 Ma) in Nihewan, Linxia Basin and Gonghe, Northern China. Then, *C. Nihowanensis* gradually evolved into *Coelodonta tologoijensis* (~ 0.75 Ma) in Western Transbaikalia, Russia, and finally became woolly rhinoceros in Europe around 0.46–0.40 Ma [2, 17, 28]. Our Bayesian analysis estimates the tMRCA of woolly rhinoceros to be around 0.57 Ma (95% CI: 0.76–0.41 Ma) (Fig. 2), while the available first occurrence of woolly rhinoceros is about 0.46 Ma [28]. Generally, the divergence time of molecular estimation is earlier than species appearance [29]. We therefore suppose that this deeply divergent clade may represent the first woolly rhinoceros population as soon as its first occurrence, later it dispersed into Asia and inhabited in Northern China at least until ~ 60,000 years ago. Alternatively, combining the phylogenetic (Figs. 2 and 3) and nucleotide diversity (Table 1) analyses, our results also strongly support an alternative scenario about the origin of woolly rhinoceros, that is, this extinct rhino possibly evolved in Northern China rather than in Europe, which needs further investigations in future studies.

### Geographic distribution model of woolly rhinoceros haplogroup C

Haplogroup C was first molecularly identified from one specimen (ND030, 39,652 ± 952 cal yBP) in Wrangel Island and considered as an isolated clade [14]. Interestingly, three of our samples fall in haplogroup C (Figs. 2 and 3; Table S2), greatly extending the known geographic range of this clade. Considering Wrangel Island connected with the continent until around 10.5 thousand years ago (ka) [30], the possible geographic distribution model of haplogroup C could be interpreted with two possibilities: (1) This haplogroup was first limited to Northern China and adjacent regions. The Wrangel Island sample (ND030), the youngest individual of haplogroup C, probably descended from a later northward dispersal of individuals from Northern China. (2) An alternative possibility is that this haplogroup persistently occupied a large area in Asia after its separation from haplogroup A (around 0.33 Ma), at least ranging from Northern China to Wrangel Island, Far Eastern Siberia.

In the network analysis that included more Chinese samples but shorter data set, six out of eight analyzed Chinese woolly rhinoceros fall in haplogroup C (Fig. 3; Table S2). Considering random sampling and the ages of the samples, haplogroup C was likely the dominating haplogroup in Northern China at least during 60,000–40,000 years ago. Although many genetic sequences have been retrieved from specimens in Wrangel Island and adjacent Siberian mainland, no other samples except the specific Wrangel Island sample ND030 (39,652 ± 952 cal yBP) has been detected belonging to haplogroup C. Compared to the above mentioned latter geographic widely distribution model, we suggest the former dispersal scenario might reflect the actual status of the Late Pleistocene woolly rhinoceros in Eastern and Northeastern Asia. The tMRCA of haplogroup C individuals is estimated at 0.20 Ma (95% CI: 0.27–0.13 Ma). We thus suggest that the genetically distinct woolly rhinoceros haplogroup C possibly inhabited Northern China since late Middle Pleistocene, which experienced a northward dispersal event with warming climates and reached its north-easternmost known occurrence, i.e., Wrangel Island, no later than approximately 40,000 years ago, that is, the Wrangel Island specimen (ND030) should originate from Northern China.

### Possible evolution center of woolly rhinoceros in Northern China

Phylogeographic investigations suggest that populations from the evolution center tend to show more abundant genetic diversity than those from migration areas [31]. Northeastern Siberia was previously proposed as an evolution center of Late Pleistocene woolly rhinoceros [18]. In this study, our findings indicate that Northern China is possibly another evolution center for Pleistocene woolly rhinoceros. Our network result (Table S2) reveals that Chinese samples fall in three out of the four identified maternal haplogroups, and a total of eight analyzed Chinese samples are assigned to seven mitochondrial haplotypes, while only five mitochondrial haplotypes identified from 18 Siberian samples (Fig. 3). It suggests that Late Pleistocene Chinese woolly rhinoceros are characterized by a high diversity of haplotypes compared with their contemporaries in Siberia. Moreover, the basal split woolly rhinoceros clade (haplogroup D) inhabited Northern China (Fig. 2). We thus emphasize this region as a key evolution center of woolly rhinoceros, and specimens from Northern China play an important role in exploring the evolution of this famous rhino species.

As above mentioned, higher level of nucleotide diversity was detected from individuals from Northern China than those from Siberia, which might imply a large population size of woolly rhinoceros that inhabited in Northern China during the Late Pleistocene. It is confirmed by numerous fossil materials unearthed from various sites in this region [6, 18, 19]. However, we also notice that the ages of the analyzed Chinese specimens are all older than 40,000 years, while most Yakutian and Chukotkan individuals from Siberia are younger than this date (Tables S1 and S2). Therefore, the difference of nucleotide diversity among these specimens also possibly reflects the loss of nucleotide diversity when woolly rhinoceros became extinct at the end of Late Pleistocene.

In addition, many molecular studies verify that the members of “*Mammuthus-Coelodonta* faunas” from Northern China are closely related to their Siberian relatives. Recent ancient genetic analyses infer that some extinct or extant species, e.g., Ovodov Horse (*Equus ovodovi*) [32, 33], steppe bison [24], roe deer (*Capreolus* spp.) [34], and red deer (*Cervus elaphus/hanglu/canadensis*) [35], dispersed between Northern China and Siberia when climate or vegetation changed. In the present study, our findings reveal that at least two out of four detected woolly rhinoceros haplogroups (i.e., B and C) are shared by samples from Northern China and Siberia (Fig. 3). As previous study by Yuan et al. [12] suggested, woolly rhinoceros specimens from Northern China had extensive interaction with the Siberian individuals. Confirming Northern China as a long-term refugium and important evolution center of woolly rhinoceros, additional genomes from different spatio-temporal specimens from this area are needed, and nuclear DNA in particular would be valuable to infer the comprehensive molecular evolution of woolly rhinoceros in the future.

## Conclusion

In the present study, we successfully generated four mitogenomes from Late Pleistocene woolly rhinoceros excavated from Northern China. Phylogenetic analyses reveal that our one sample forms a distinct divergent clade, which possibly representing the earliest split clade almost as soon as the first occurrence of this species. Our other three samples cluster within a separate clade, initially represented by one sample from Wrangel Island. Our findings imply an ancient northward dispersal of this clade and greatly extend its known geographic range. Moreover, the available molecular data indicates that Chinese woolly rhinoceros shows a higher genetic diversity than its Siberian relatives. Taken together numerous fossil materials, our genetic data infer that Northern China plays an important role in the evolution of Pleistocene woolly rhinoceros.

### Methods

#### Information of samples

In total, we sampled 16 woolly rhinoceros specimens from Xinbarhu Left Banner, Wushen Banner, Wulanmulun Paleolithic site, and Salawusu in Inner Mongolia Autonomous Region; Huan County and Zhengning County in Gansu Province; and Qinggang County, Zhaoyuan County in Heilongjiang Province, respectively (Table S1). These sites represent the main distribution area of Late Pleistocene woolly rhinoceros in Northern China (Fig. 1). Mitochondrial genomes were successfully retrieved from four out of 16 samples (CADG739, CADG744, CADG900 and CADG912), that is, > 75% sequence coverage of the woolly rhinoceros mitochondrial reference (GenBank No. FJ905813). We subsequently sent these four samples to Beta Analytic Testing Laboratory in USA for radiocarbon dating, and the ^14^ C dates were calibrated with IntCall20 northern hemisphere radiocarbon calibration curve [36].

### DNA extraction and library preparation

DNA extraction and library preparation were performed in a dedicated laboratory for ancient samples at China University of Geosciences (Wuhan). Firstly, we removed the surface of the samples to exclude the potential contamination. The inner part of each sample was ground to powder. Approximately 100–150 mg teeth or bone powder of each sample was digested in 4.5 mL of EDTA (0.5 M, pH = 8) and 0.06 mL of Proteinase K (20 mg/mL) for 16 h in a rotating hybridization oven at 37℃. Next, the digest was centrifuged at 7,000 rpm for 10 min. The supernatant was then transferred into an ultrafiltration tube (Millipore, Germany) and condensed to about 100 µL at 7,000 rpm. Finally, the condensed product was purified with MinElute PCR Purification Kit (Qiagen, Germany) and eluted in 50 µL EB buffer according to the manufacturer’s instructions.

For each sample, 20 µL DNA extraction was used to construct double-stranded DNA library following the protocol by Meyer and Kircher [37]. The reaction system of the blunt-end repair contains NEB buffer (New England Biolabs, UK), dNTP (Tiangen, China), BSA (New England Biolabs, UK), T4 PNK (New England Biolabs, UK) and T4 Polymerase (New England Biolabs, UK). The original concentration of the adapters is 100 µM and a 1:20 diluted adapter mixture diluted with Quick Ligase buffer (New England Biolabs, UK) was used for the Adapter Ligation step, Isothermal buffer (New England Biolabs, UK) was used during Adapter Fill-in. The libraries were incubated at 37 ℃ for 20 min, and then heat-inactivated at 80 ℃ for 20 min. Indexing PCR amplifications were performed with using Q5 High-Fidelity DNA Polymerase (New England Biolab, UK) using dual Primers (P5 and P7 for Illumina sequencing platform). All the purification procedures were performed with the MinElute spin columns following the manufacturer’s protocols. In addition, both extraction and library blanks were set up to detect the potential contamination. The sequencing was performed on an Illumina Hiseq×10 platform using a 150 bp paired-end double-indexed protocol.

### Sequencing data processing

For each sample, we prepared 1–9 libraries (Table S1). Adapters of raw Illumina sequencing data were trimmed, reads shorter than 30 bp were discarded, and the trimmed paired-end reads were merged with fastp 0.23.2 [38]. The merged reads were subsequently mapped to a complete mitochondrial genome of woolly rhinoceros (GenBank No. FJ905813) using the Burrows-Wheeler Aligner 0.6.2 with the ‘aln’ algorithm [39]. The reference was modified by adding 30 bp from the opposite edge before mapping to improve the final mitochondrial consensus sequences. The aligned sequences were filtered for mapping quality 30 using ‘view’ and sorted using ‘sort’ in SAMtools 0.1.19 [40]. Duplicate sequences generated during PCR amplification were removed by ‘rmdup’ in SAMtools. The final mitochondrial consensus sequences were generated in Geneious 10 (http://www.geneious.com/), and positions with at least 2× coverage and a 75% consensus threshold were taken into consideration. The coverage of consensus sequences was calculated using Qualimap 2.2.2 [41] and the ancient DNA damage parameters were estimated using MapDamage 2.0 [42].

We also downloaded the sequencing raw data of 14 woolly rhinoceros individuals from European Nucleotide Archive (ENA) (Table S2), and the sequencing reads processing was carried out as above described.

### Phylogenetic analyses

We performed phylogenetic analysis in BEAST 1.8.4 [43] using our four newly obtained mitochondrial genomes and the 14 Siberian woolly rhinoceros sequences from Lord et al. [14] (Data set 1; Table S2), and selected Merck’s rhinoceros (GenBank No. KX646743) as outgroup. The sequences were aligned with MAFFT 7.471 [44] algorithm in the Cipres Science Gateway 3.3 [45]. Then, we deleted all gaps and missing data and obtained a final length of a data set of 11,704 bp. To calibrate the evolutionary rate, we selected the divergence time between Merck’s rhinoceros and woolly rhinoceros (5.50 ± 0.70 Ma) [15] and the available median calibrated radiocarbon ages of some samples (Table S2) as calibration points. As to the samples whose dates are beyond the calibration range and no strata age, i.e., CADG744, CADG900 and CADG912, we estimated the date of each infinite sample in a separate run, and the median age from BEAST estimation was considered as sample age to calibrate the Bayesian tree (Table S2). The best evolutionary model ‘HKY + F + I’ was chosen for BEAST analysis using in jModelTest 2.1.9 [46] under the Bayesian Inference Criterion. A constant population size, together with a strict molecular clock with a uniform distribution and initial rate of 2.34 × 10^− 8^ substitutions/site/year [14], was specified as tree prior. The Markov Chain Monte Carlo (MCMC) was run for 10 million generations, sampling every 1,000 generations. We checked run convergence in Tracer 1.7.1 [47], by making sure all run parameters had an effective sample sizes (ESS) > 200. TreeAnnotator 1.8.3 (https://beast.community/treeannotator) was used to remove a burn-in of 20% from the tree files. The phylogenetical trees were then visualized using FigTree 1.4.4 (http://tree.bio.ed.ac.uk/software/figtree).

To further investigate the biogeographical structure of Late Pleistocene Chinese woolly rhinoceros, we reconstructed a median-joining network using 563 bp *cyt b* sequences in Popart 1.7 (http://popart.otago.ac.nz), which contains more Chinese specimens (Data set 2; Table S2) than those in BEAST analysis.

### Nucleotide diversity analysis

We divided the Siberian woolly rhinoceros specimens into two parts, i.e., Yakutian and Chukotkan individuals. Then we compared the nucleotide diversity of the Late Pleistocene woolly rhinoceros from Northern China (N = 4), Yakutia (N = 8) and Chukotka regions (N = 9) in MEGA 7 [48], using 11,609 bp mitogenomic sequences as all gaps, missing data and degenerate sites were excluded (Data set 3; Table S2).

### Electronic supplementary material

Below is the link to the electronic supplementary material.

Additional file 1: **Table S1**. The information of woolly rhinoceros specimens and the retrieved mitogenomes in this study. **Table S2.** Near-Complete mitochondrial sequences or *cyt b* genes of woolly rhinoceros used in this study. **Figure S1.** Estimated endogenous fragment length distributions of woolly rhinoceros individuals analyzed in this study. **Figure S2.** Cytosine deamination frequency inferred from woolly rhinoceros individuals analyzed in this study. **Figure S3.** Maximum Likelihood phylogenetic tree of woolly rhinoceros in MEGA 7 using 11,704 bp homologous mitogenomes.

## Data Availability

The GenBank accession numbers for the four mitochondrial genomes reported in this paper are OP803072-5, respectively.
