# Supplementary material for: Ancient mitogenomes reveal a high maternal genetic diversity of Pleistocene woolly rhinoceros in Northern China
Source: BMC Ecol Evol. 2023 Sep 26;23:56. doi: 10.1186/s12862-023-02168-0 (PMC10521388; doi:10.1186/s12862-023-02168-0)
Supplement: Supplementary file 1 — Additional file 1: Table S1. The information of woolly rhinoceros specimens and the retrieved mitogenomes in this study. Table S2. Near-Complete mitochondrial sequences or cyt b genes of woolly rhinoceros used in this study. Figure S1. Estimated endogenous fragment length distributions of woolly rhinoceros individuals analyzed in this study. Figure S2. Cytosine deamination frequency inferred from woolly rhinoceros individuals analyzed in this study. Figure S3. Maximum Likelihood phylogenetic tree of woolly rhinoceros in MEGA 7 using 11,704 bp homologous mitogenomes. [file 12862_2023_2168_MOESM1_ESM.docx]

**Supplementary material**

**Table S1.** The information of woolly rhinoceros specimens and the retrieved mitogenomes in this study.

| Sample ID | Geographical location | ^14^C date (yBP) | Number of libraries | Average read depth (×) | Length (bp) | Average fragment length (bp) |
| --- | --- | --- | --- | --- | --- | --- |
| CADG739 | Qinggang County, Heilongjiang Province | 41,080 ± 260 (Beta - 588745) | 9 | 3.31 | 12,660 | 56 |
| CADG744 | Xinbarhu Left Banner, Inner Mongolia | > 43,500 (Beta - 588746) | 3 | 20.60 | 16,233 | 74 |
| CADG799 | Huan County, Gansu Province | – | 1 | – | – | – |
| CADD801 | Huan County, Gansu Province | – | 1 | – | – | – |
| CADG802 | Huan County, Gansu Province | – | 1 | – | – | – |
| CADG814 | Zhengning County, Gansu Province | – | 1 | – | – | – |
| CADG818 | Zhengning County, Gansu Province | – | 1 | – | – | – |
| CADG863 | Wushen Banner, Inner Mongolia | – | 1 | – | – | – |
| CADG867 | Wulanmulun site, Inner Mongolia | – | 1 | – | – | – |
| CADG894 | Salawusu site, Inner Mongolia | – | 1 | – | – | – |
| CADG900 | Salawusu site, Inner Mongolia | > 43,500 (Beta - 632231) | 5 | 10.86 | 15,883 | 56 |
| CADG912 | Salawusu site, Inner Mongolia | > 43,500 (Beta - 632232) | 4 | 6.48 | 15,471 | 66 |
| CADG1020 | Qinggang County, Heilongjiang Province | – | 1 | – | – | – |
| CADG1043 | Zhaoyuan County, Heilongjiang Province | – | 1 | – | – | – |
| CADG1044 | Zhaoyuan County, Heilongjiang Province | – | 1 | – | – | – |
| CADG1045 | Zhaoyuan County, Heilongjiang Province | – | 1 | – | – | – |

**Table S2.** Near-Complete mitochondrial sequences or *cyt b* genes of woolly rhinoceros used in this study.

| Accession No. | Sample ID | ^14^C date (cal yBP) | Geographic region | Data set 1 | Data set 2 | Data set 3 | Haplogroup | Reference |
| --- | --- | --- | --- | --- | --- | --- | --- | --- |
| OP803072 | CADG739 | 43,942 ± 593 | Northern China | √ | √ | √ | C | In this study |
| OP803073 | CADG744 | 62,453* | Northern China | √ | √ | √ | D | In this study |
| OP803074 | CADG900 | 51,721* | Northern China | √ | √ | √ | C | In this study |
| OP803075 | CADG912 | 59,810* | Northern China | √ | √ | √ | C | In this study |
| SAMEA6246862 | ND003 | 30,789 ± 32 | Chukotka | √ | √ | √ | A | [1] |
| SAMEA6246863 | ND004 | 43,560 ± 745 | Chukotka | √ | √ | √ | A | [1] |
| SAMEA6246864 | ND008 | 32,757 ± 830 | Chukotka | √ | √ | √ | A | [1] |
| SAMEA6246865 | ND010 | 31,511 ± 282 | Chukotka | √ | √ | √ | A | [1] |
| SAMEA6246866 | ND012 | 34,761 ± 673 | Chukotka | √ | √ | √ | B | [1] |
| SAMEA6246867 | ND014 | 40,934 ± 1280 | Yakutia | √ | √ | √ | A | [1] |
| SAMEA6246868 | ND015 | 30,611 ± 338 | Yakutia | √ | √ | √ | A | [1] |
| SAMEA6246869 | ND030 | 39,652 ± 952 | Wrangel Island | √ | √ | √ | C | [1] |
| SAMEA6246870 | ND031 | 17,850 ± 215 | Chukotka | √ | √ | √ | B | [1] |
| SAMEA6246871 | ND035 | 18,527 ± 171 | Chukotka | √ | √ | √ | B | [1] |
| SAMEA6246872 | ND036 | 48,090 ± 1909 | Chukotka | √ | √ | √ | A | [1] |
| SAMEA6246873 | ND041 | 14,161 ± 210 | Yakutia | √ | √ | √ | A | [1] |
| SAMEA6246874 | ND045 | 36,445** | Yakutia | √ | √ | √ | A | [1] |
| SAMEA6246875 | DS253 | 14,393 ± 295 | Yakutia | √ | √ | √ | B | [1] |
| FJ905813 | – | – | Yakutia | × | √ | √ | B | [2] |
| NC_012681 | – | – | Yakutia | × | √ | √ | B | [2] |
| MK909152 | MK019 | – | Yakutia | × | √ | √ | A | [3] |
| DQ318533 | PIN3342-103 | > 49,000 | Yakutia | × | √ | × | A | [4] |
| GU371439 | HS14 | About 39,000^#^ | Northern China | × | √ | × | C | [5] |
| GU371440 | HS12 | 39625 ± 250^#^ | Northern China | × | √ | × | B | [5] |
| JQ974920 | SL1 | About 42,000^#^ | Northern China | × | √ | × | C | [5] |
| JQ974921 | SL4 | 42230 ± 370^#^ | Northern China | × | √ | × | C | [5] |

* The median ages estimated using BEAST in this study.

** Estimated age in Lord et al. [1].

^#^ ^14^C age or stratigraphic chronology according to Yuan et al. [5].

**Reference**

[1] Lord E, Dussex N, Kierczak M, Diez-del-Molino D, Ryder OA, Stanton DWG, Gilbert MTP, Sanchez-Barreiro F, Zhang G, Sinding MS, Lorenzen ED, Willerslev E, Protopopov A, Shidlovskiy F, Fedorov S, Bocherens H, Nathan SKSS, Goossens B. Pre-extinction demographic stability and genomic signatures of adaptation in the woolly rhinoceros. Curr Biol. 2020; 30: 3871-3879.

[2] Willerslev E, Gilbert MTP, Binladen J, Ho SYW, Campos PF, Ratan A, Tomsho LP, da Fonseca RR, Sher A, Kuznetsova TV, Nowak-Kemp M, Roth TL, Miller W, Schuster SC. Analysis of complete mitochondrial genomes from extinct and extant rhinoceroses reveals lack of phylogenetic resolution. BMC Evol Biol. 2009; 9: 1-11.

[3] Margaryan A, Sinding MHS, Liu S, Vieira FG, Chan YL, Nathan SK, Moodley Y, Bruford MW, Gilbert MTP. Recent mitochondrial lineage extinction in the critically endangered Javan rhinoceros. Zool J Linn Soc*.* 2020; 190: 372-383.

[4] Binladen J, Wiuf C, Gilbert MTP, Bunce M, Barnett R, Larson G, Greenwood AD, Haile J, Ho SYW, Hansen AJ, Willerslev E. Assessing the fidelity of ancient DNA sequences amplified from nuclear genes. Genetics 2006; 172: 733-741.

[5] Yuan J, Sheng G, Hou X, Shuang X, Yi J, Yang H, Lai X. Ancient DNA sequences from *Coelodonta antiquitatis* in China reveal its divergence and phylogeny. Sci China (Earth Sci.) 2014; 57: 388-396.


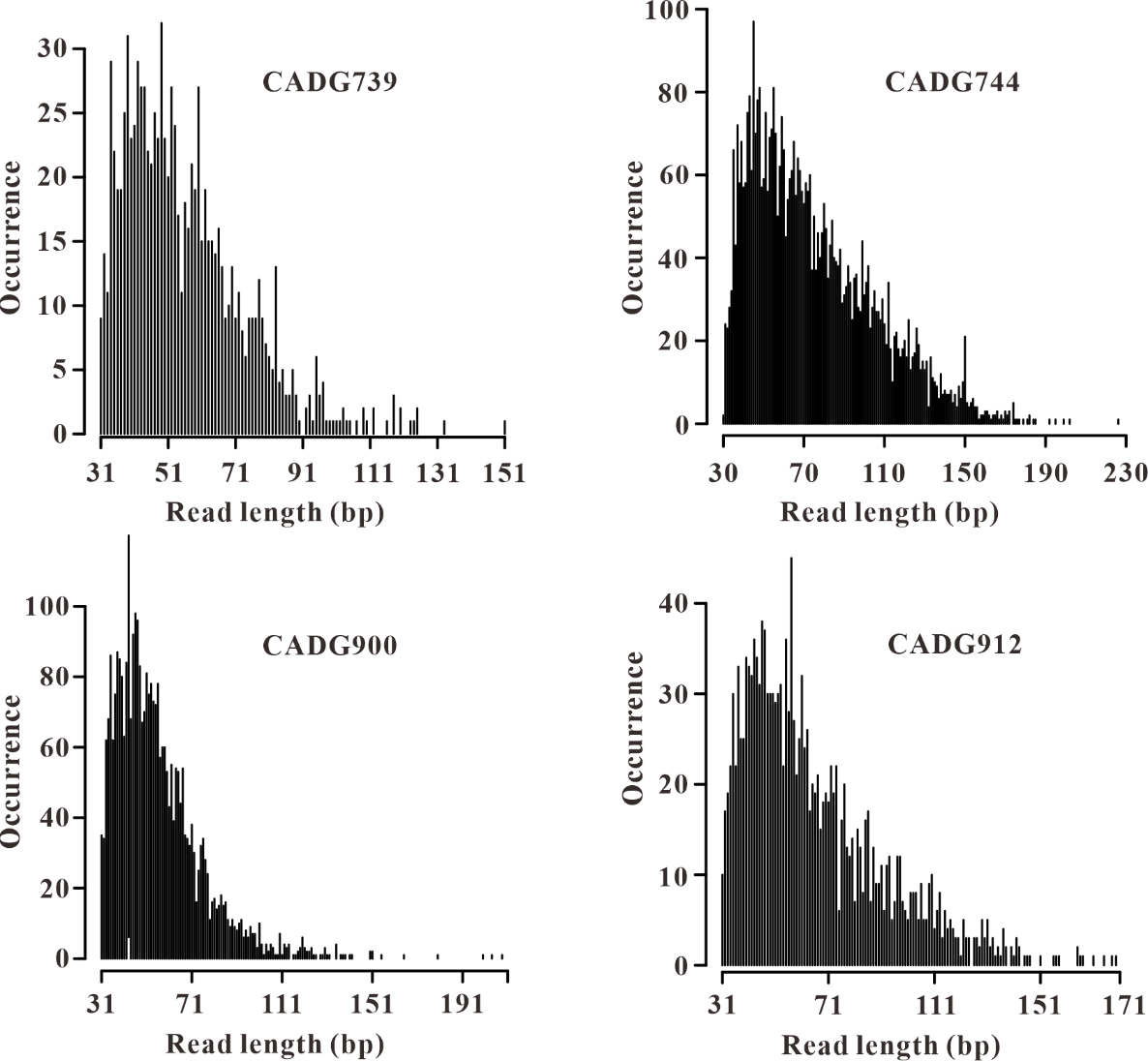


**Figure S1.** Estimated endogenous fragment length distributions of woolly rhinoceros individuals analyzed in this study.


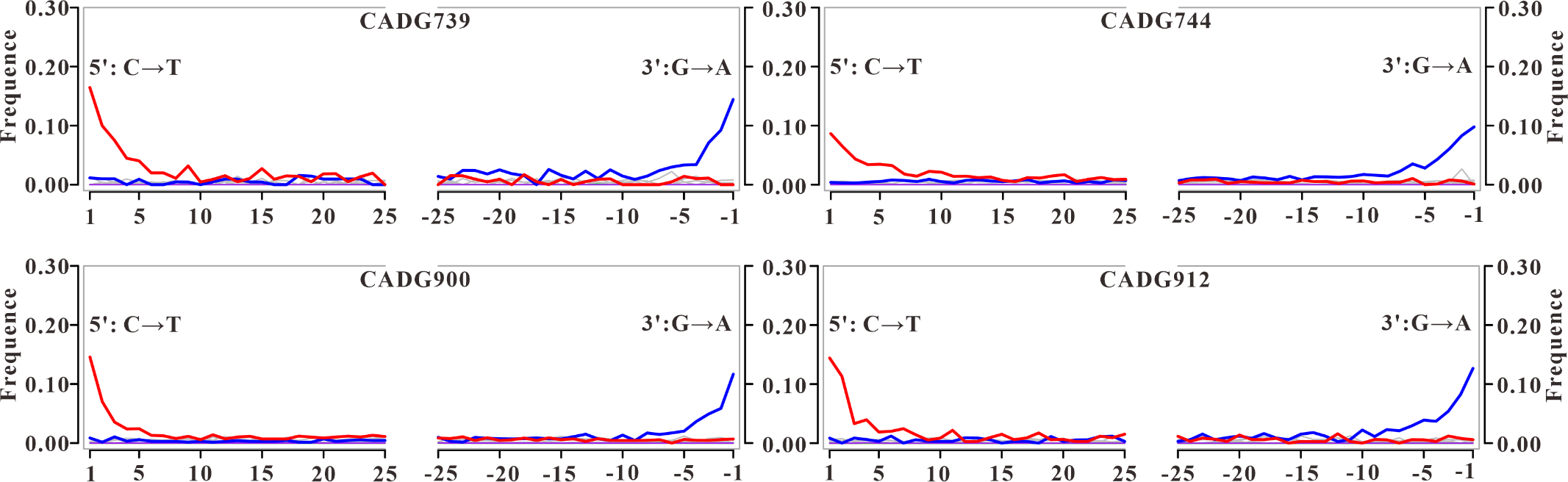


**Figure S2.** Cytosine deamination frequency inferred from woolly rhinoceros individuals analyzed in this study.


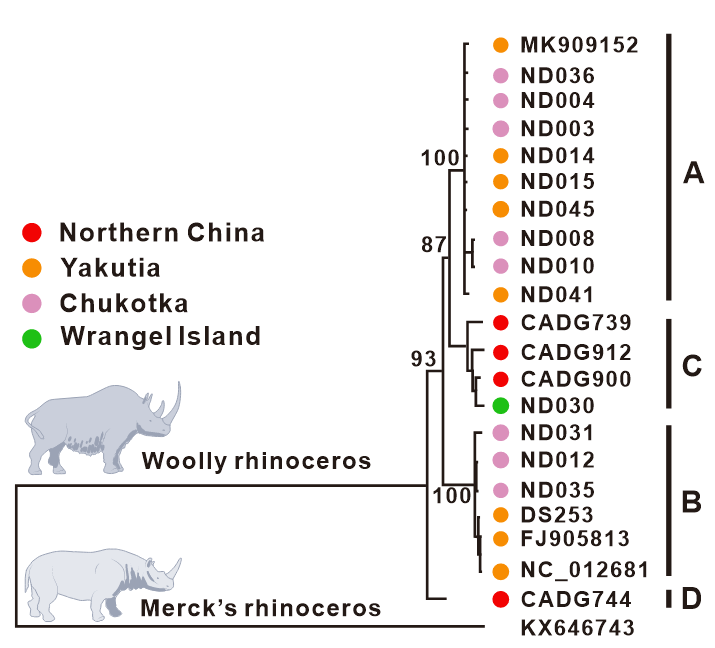


**Figure S3.** Maximum Likelihood phylogenetic tree of woolly rhinoceros in MEGA 7 using 11,704 bp homologous mitogenomes. Merck’s rhinoceros was selected as outgroup. Branch labels show the bootstrap values (>70%) derived from 1000 replications.
